# Closing the gap: advancing implementation science through training and capacity building

**DOI:** 10.1186/s13012-024-01371-x

**Published:** 2024-07-03

**Authors:** Ross C. Brownson, Leopoldo J. Cabassa, Bettina F. Drake, Rachel C. Shelton

**Affiliations:** 1 Prevention Research Center, Brown School at Washington University in St. Louis, One Brookings Drive, Campus, Box 1196, St. Louis, MO 63130 United States; 2 Department of Surgery, Division of Public Health Sciences, and Alvin J. Siteman Cancer Center, Washington University School of Medicine, Washington University in St. Louis, St. Louis, MO 63130 United States; 3 Center for Mental Health Services Research, Brown School, Washington University in St. Louis, St. Louis, MO 63130 63130 United States; 4 Department of Sociomedical Sciences, Columbia University Mailman School of Public Health, New York, NY 10032 United States

**Keywords:** Capacity building, Competencies, Equity, Implementation science, Sustainability, Training

## Abstract

In their article on “Navigating the Field of Implementation Science Towards Maturity: Challenges and Opportunities,” Chambers and Emmons describe the rapid growth of implementation science along with remaining challenges. A significant gap remains in training and capacity building. Formats for capacity building include university degree programs, summer training institutes, workshops, and conferences. In this letter, we describe and amplify on five key areas, including the need to (1) identify advanced competencies, (2) increase the volume and reach of trainings, (3) sustain trainings, (4) build equity focused trainings, and (5) develop global capacity. We hope that the areas we highlight will aid in addressing several key challenges to prioritize in future efforts to build greater capacity in implementation science.

In their insightful editorial, Chambers and Emmons provide a brief history of implementation science (IS) and opportunities to expand the reach and impact of our field [1]. Of particular salience is the section on advances in capacity building needed for IS. Chambers and Emmons note several gaps in our training and mentoring, including the need to move beyond the”100-level courses” and the imperative to more fully sustain training and capacity building efforts.

Recent reviews of initiatives to build capacity in IS have shown a growing number of types of training and mentoring opportunities across 13 countries [2]. Capacity building for IS occurs in multiple formats including university degree programs, summer training institutes, workshops, and conferences [3, 4].

To amplify these needs, we offer the following questions and initial thoughts on addressing these critical gaps and needs for capacity building.

1. What are high-priority, advanced competencies in IS?
2. How might we increase the volume and reach of training and capacity building?
3. How do we better sustain training and capacity building programs?
4. How do we more fully integrate equity in IS training programs?
5. What are opportunities for training and capacity building across the globe?

## Articulate advanced competencies

**Fig. 1:**
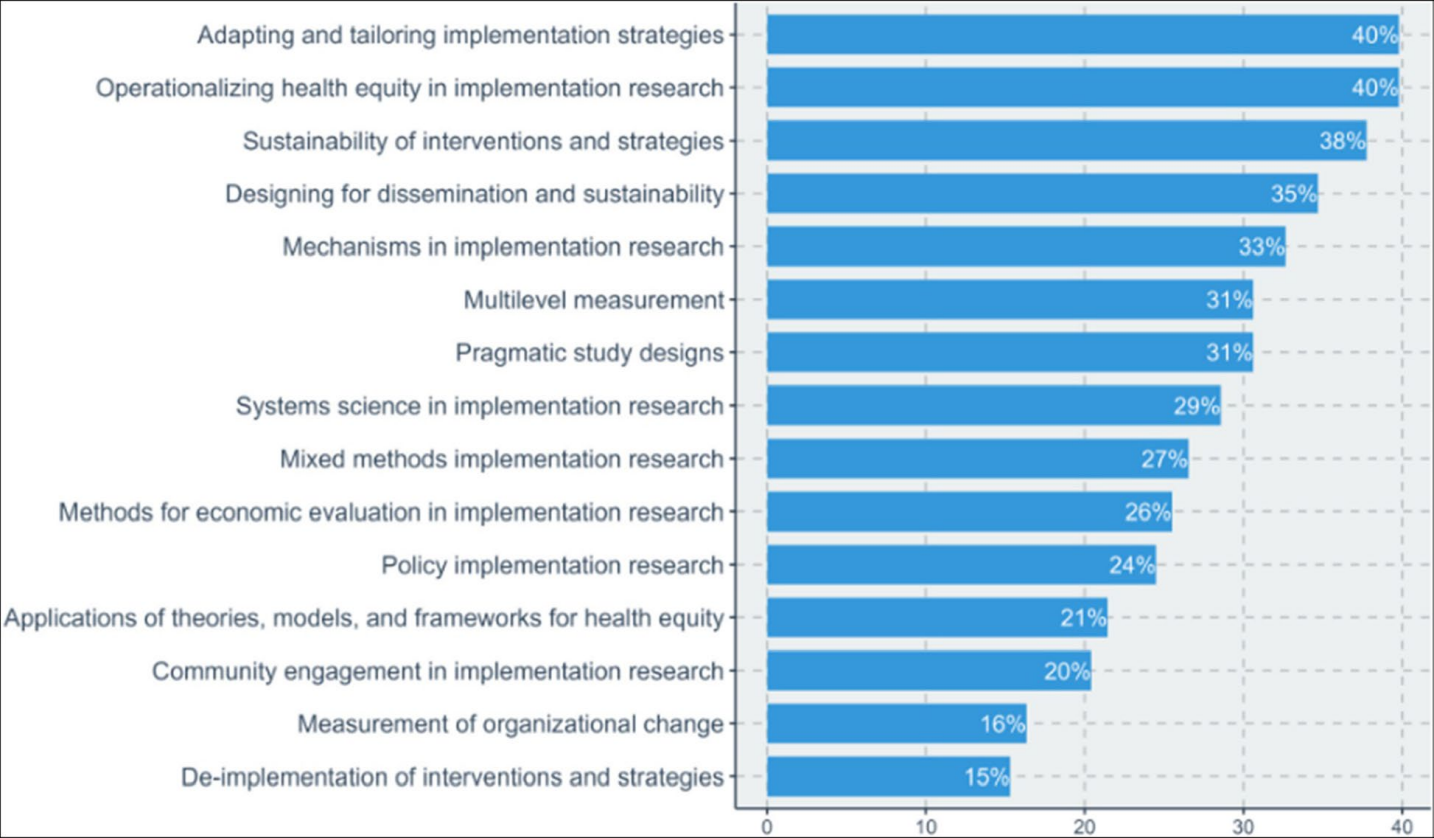
*The percentages represent the frequency by which respondents (*n* = 97) selected each skill as one of their top 5 priorities

As we gain greater understanding and capacity in beginning and intermediate skills and competencies in IS, the need for the development of more advanced skills has become apparent [5]. To identify a set of priority advanced skills, we conducted a three-part project in late 2023. In part one, we mapped existing competencies from previous studies [6, 7] and the latest edition of a foundational textbook in IS [5]. This mapping identified an initial set of advanced competencies. In part two, a set of 20 experts in IS reviewed competencies and suggested edits to wording and additions. In part three, we conducted a survey of 97 experts in IS to prioritize 15 advanced competencies (Fig. 1). Additionally, in depth, evidence-informed mentoring enhances productivity [8] and should be a core activity for applying these competencies. Evidence-informed mentoring follows a systematic approach that applies mentoring frameworks, training of mentors and mentees, continuous improvement, and evaluation of the mentoring experience and outcomes [9, 10]. Excellent, no-cost resources are available to enhance mentoring capacity [11].

## Increase the volume and reach of trainings

The supply of trainings in IS lags far behind the demand. For example, among six major IS training programs for researchers in the United States [12–17] from 2011 through 2023, there was a total of 2,080 applicants to these programs and 575 acceptances (a 28% acceptance rate) (personal communication, Sarah Bernal, January 8, 2024). The acceptance rate is slightly higher for trainings organized by universities (35%) compared with those organized by US National Institutes of Health (25%). Most US trainings have focused on mental health and cancer [1], illustrating the need to broaden coverage. Notably, there are also few training programs focused on implementers—often these are practitioners who are users of research who need to learn a set of skills (e.g., how to assess local context, how to adapt interventions) [18, 19]. MOOCs (massive open online courses) show promise for enhancing the reach of IS trainings for both researchers and implementers [20].

## Understand how to sustain trainings

Our understanding of the sustainability of interventions and implementation strategies has grown considerably over the past 15 years with a rapid increase in the literature and practical tools [21]. However, knowledge of the sustainability of IS training is far less advanced. There is a need for more research on determinants and practical tools for sustaining trainings in IS. Institutional policies likely play essential roles by providing core funding for training, better rewarding mentoring in academic promotion criteria, and making IS a core element of coursework (with core competencies) in graduate-level public health, clinical, and social service training programs.

## Build equity focused trainings

While our field is raising the visibility and priority of equity in IS [22, 23], there are few training programs that focus at the intersection of health equity and IS that build competence in each discipline [6]. A vision for such training includes multiple parts: 1) how a training program reaches diverse scholars and faculty, 2) how training is delivered to reach broad audiences (including those outside health sectors), 3) whether equity is featured as an explicit part of core competencies, 4) how participatory research approaches are included in training, and 5) how best to evaluate progress in achieving equity as a core competency.

## Develop global capacity

The reach of IS training programs falls far short of meeting the global demand [2, 24], particularly in low-and middle-income countries (LMICs) (e.g., countries on the African continent) that have the highest preventable burden of disease [25]. Since nearly all competencies for IS have been developed in high-income countries, there is a need for aligning skill sets to LMIC contexts [26]. We should develop cross-national evaluation approaches, including common metrics—for example, one could envision a core set of competencies and additional competencies that are tailored to local context. Capacity building should build on innovations in LMICs, which can be higher than in high resource settings [27]. Country-specific [28] and regional [29] IS training programs will allow us to eventually develop a menu of training approaches.

The growing literature on training and capacity building in IS illustrates where progress has been made, yet critical gaps and opportunities remain [1]. It is our hope that the areas we briefly highlight will aid in addressing several key challenges to prioritize in future work.

## Data Availability

The data analyzed during the current study are available from the corresponding author upon reasonable request.

## Abbreviations

IS: Implementation science
LMICs: Lower and middle income countries
MOOCs: Massive Open Online Courses
